# Hybrid Machine Learning-Based Fault-Tolerant Sensor Data Fusion and Anomaly Detection for Fire Risk Mitigation in IIoT Environment

**DOI:** 10.3390/s25072146

**Published:** 2025-03-28

**Authors:** Jayameena Desikan, Sushil Kumar Singh, A. Jayanthiladevi, Shashi Bhushan, Vinay Rishiwal, Manish Kumar

**Affiliations:** 1 Department of Computer Engineering, Marwadi University, Rajkot 360003, Gujarat, India; 2 Computer & Information Sciences Department, Universiti Teknologi PETRONAS, Bandar Seri Iskandar 32610, Malaysia; 3 Department of CSIT, MJP RohilKhand University, Bareilly 243006, Uttar Pradesh, India

**Keywords:** IIoT, faulty sensors, anomaly detection, Dempster–Shafer, machine learning, sensor fusion

## Abstract

In the oil and gas IIoT environment, fire detection systems heavily depend on fire sensor data, which can be prone to inaccuracies due to faulty or unreliable sensors. These sensor issues, such as noise, missing values, outliers, sensor drift, and faulty readings, can lead to delayed or missed fire predictions, posing significant safety and operational risks in the oil and gas industrial IoT environment. This paper presents an approach for handling faulty sensors in edge servers within an IIoT environment to enhance the reliability and accuracy of fire prediction through multi-sensor fusion preprocessing, machine learning (ML)-driven probabilistic model adjustment, and uncertainty handling. First, a real-time anomaly detection and statistical assessment mechanism is employed to preprocess sensor data, filtering out faulty readings and normalizing data from multiple sensor types using dynamic thresholding, which adapts to sensor behavior in real-time. The proposed approach also deploys machine learning algorithms to dynamically adjust probabilistic models based on real-time sensor reliability, thereby improving prediction accuracy even in the presence of unreliable sensor data. A belief mass assignment mechanism is introduced, giving more weight to reliable sensors to ensure they have a stronger influence on fire detection. Simultaneously, a dynamic belief update strategy continuously adjusts sensor trust levels, reducing the impact of faulty readings over time. Additionally, uncertainty measurements using Hellinger and Deng entropy, along with Dempster–Shafer Theory, enable the integration of conflicting sensor inputs and enhance decision-making in fire detection. This approach improves decision-making by managing sensor discrepancies and provides a reliable solution for real-time fire predictions, even in the presence of faulty sensor readings, thereby mitigating the fire risks in IIoT environments.

## 1. Introduction

Predicting fires with a high degree of certainty remains particularly difficult, especially in the oil and gas IIoT environment. Due to the unpredictable nature of climatic conditions worldwide, early fire forecasting has become increasingly challenging and critical for many sectors within the oil and gas industry. Ensuring the safety of workers and industrial equipment remains a top priority in these industries. Fire risk reduction is a critical aspect of industrial safety, particularly in high-risk environments such as oil and gas facilities. The presence of flammable materials, high-pressure systems, and extreme operational conditions make fire hazards a major concern. Effective fire prevention depends on understanding the fundamental principles of fire formation, which include the fire triangle: heat, fuel, and oxygen, all of which are necessary for combustion in the oil and gas industry. By identifying and monitoring these key fire elements through sensor-based technologies, early warning systems can significantly reduce the likelihood of uncontrolled fires. Strategies for mitigating advanced fire risks not only protect human lives and industrial assets but also help to ensure regulatory compliance and environmental sustainability. The rapid growth of IoT applications has significantly increased reliance on sensor data for critical decision-making processes, including fire detection and prevention systems in remote oil and gas environments. However, the quality of sensor data and the deployment of these systems for fire prediction heavily influence their accuracy and reliability. Fire sensors are devices that detect early warning signs of fire, such as smoke, heat, gas leaks, or flames. Instead of relying on just one type of sensor, the proposed approach combines multiple sensors, such as RGB sensors, infrared sensors, smoke detectors, gas sensors, and flame sensors, to improve accuracy.

**Figure 1:**
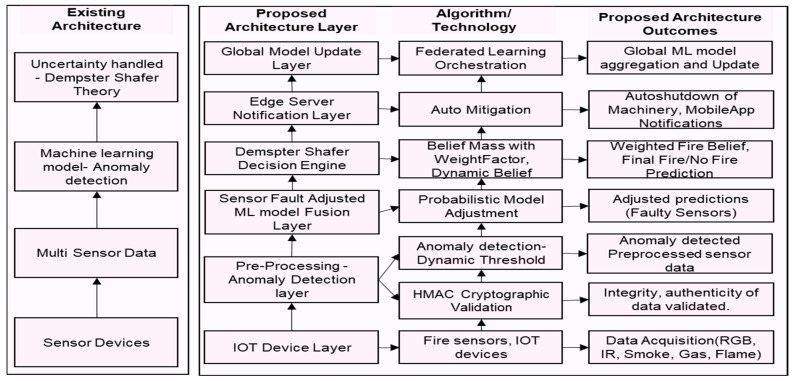
Existing and proposed architecture for fire prediction with anomaly detection.

Sensor faults, such as bias, drift, or noise, can severely impact the performance of anomaly detection algorithms. Sensor data pre-processing is important to address faulty sensor data and enhance its quality and reliability in IoT environments, where sensor data are often noisy and inconsistent [1]. Misclassification or misinterpretation of sensor data can occur, directly affecting how well fire predictions perform. Therefore, faulty sensors should be accounted for when making fire predictions. Different sensors detect distinct fire-related parameters. By combining them, a broader range of fire indicators can be captured, reducing the risk of missing key fire warning signs. According to existing research studies, the challenge is to fuse and analyze sensor data holistically, and faulty sensors must also be considered [2,3]. Past research studies have combined data from multiple sensors and detected changes in the data distribution using a fire detection mechanism based on multi-sensory data fusion [4]. Fire sensors generate complex data patterns influenced by fire type, environmental conditions, and noise from non-fire events. To address these complexities and provide accurate detection while reducing false alarms, advanced data-driven methods must be employed. These methods should distinguish fires from non-fire events and adapt dynamically to new environments and fire types without requiring extensive manual calibration. Past research has integrated data from IoT sensors with machine learning techniques to carry out fault diagnosis across different systems [5,6]. A fault diagnosis algorithm has been proposed based on the Dempster–Shafer theory of evidence fusion and deep convolutional neural networks [7,8,9]. Existing and proposed architecture for fire prediction with anomaly detection is shown in Figure 1.

### 1.1. Motivation

The motivation behind this research stems from the increasing need for accurate and reliable fire detection systems in high-risk environments like oil and gas industries, where safety is of high importance. Despite advancements in sensor-based technologies, predicting fires remains difficult due to sensor faults, environmental uncertainty, and conflicting data from multiple sensors. The challenges in combining data from various sources without introducing errors or missing critical fire-related indicators create a significant gap in existing systems. Present fire detection methods are often hindered by faulty sensor data, and the potential for false alarms or missed fire events leads to safety risks and operational inefficiencies. Addressing these issues requires a system capable of processing complex sensor data, identifying anomalies, and mitigating faulty sensor impacts. Hence, the motivation for this research is to develop an innovative approach that improves fire prediction systems by reducing the impact of faulty sensors and enhancing the overall reliability of fire detection processes.

### 1.2. Contribution

The main contribution of this research is developing a novel multi-sensor data fusion approach that effectively handles faulty sensor data in the oil and gas IIoT environment for improved fire prediction accuracy. This paper proposes the integration of multiple sensor types, such as RGB sensors, infrared sensors, smoke detectors, gas sensors, and flame sensors, to increase detection sensitivity and reduce the risk of false fire alarms. By leveraging machine learning algorithms and probabilistic adjustments, the proposed system dynamically updates sensor data to mitigate the effect of faulty sensors. This results in more accurate and timely fire predictions, even in remote environments.

Specifically, the contributions of this research include the following:

- Multi-sensor fusion that accounts for faulty sensor data.
- The use of machine learning algorithms and probabilistic adjustments based on model outputs and sensor status refine and improve fire detection accuracy and reliability.
- DSET updates belief masses dynamically, mitigating faulty sensor impact through adaptive weighting.
- Handling faulty sensor data in edge computing results in immediate fire detection, enhancing security, and reducing latency compared to cloud computing.
- Integrated sensor inputs, including faulty sensor data, ensure that no fire-related signal parameters are missed or disregarded.

### 1.3. Organization

The paper is organized as follows: Section 2 reviews existing research studies related to faulty sensors, including sensor pre-processing; hybrid machine learning algorithms handling faulty sensors, fire, and fire-related sensors in oil and gas industries; and the Dempster–Shafer evidence theory considering faulty sensors. Section 3 presents the proposed architecture for hybrid machine learning-based fault-tolerant sensor data fusion and anomaly detection for fire risk mitigation in IIoT environments. This section includes a technical flow overview and problem formulation. Section 4 discusses the results and performance of the proposed solution, highlighting its effectiveness in comparison to existing methods. Section 5 compares the results of the proposed approach with the existing research studies, demonstrating improvements in the accuracy, reliability, and timeliness of fire prediction. Section 6 concludes the paper with a summary of the findings and suggestions for future research in this area.

## 2. Related Work

In this section, we discussed existing research studies in detail and showed how we can address existing research study’s challenges with the proposed work. Also, we discussed the significance of the proposed work.

### 2.1. Existing Research Studies

Su, Z. et al. (2020) made an advanced fault diagnosis framework based on singular value manifold features (SVMFs), optimized support vector machines (SVMs), and multi-sensor information fusion. SVMFs that encode faults with singular spectra manifold topology features and SVM parameters are optimized with an enhanced fruit fly algorithm. The strength and accuracy of fault detection for multi-sensor data fusion have been improved by the Dempster–Shafer theory [10]. In unmanned surface vehicles, Qiao, S. et al. (2023) incorporate fuzzy Dempster–Shafer evidence theory for data fusion from multi-sensors. To reconcile sensor information with conflicts more precisely, researchers have developed new measures of belief divergence and fuzzy support. This method accommodates many types of sensors and allows better fusion of data for marine navigation [11]. Li, S. et al. (2017) integrated deep convolutional neural networks with Dempster–Shafer evidence theories for bearing-fault diagnosis using IDSCNN. The distance matrix and Gini-Index-enhanced evidence integration were used to refine evidence fusion, and the model configurations in the presence of conflicting data and varying load conditions were made [12]. Wang, Z. et al. (2018) developed a multi-sensor data fusion method to increase the efficiency of fault diagnosis in challenging systems. They refined this method to address the conflicting data from the sensors [13]. Attarha et al. (2024) unveiled AssureSense, a cutting-edge fault detection scheme that enhances the capabilities of the Dempster–Shafer theory by incorporating a systematic data-driven feature extraction method, TsAssure. The latter allows for improved fault detection across all levels in sensor data and helps locate the most inconspicuous hidden faults. TsAssure extracts meaningful temporal, local, and spatial features from sensor measurements against interdependent sensors, delivering a prime advantage in understanding sensor behavior [14]. Wang et al. (2019) demonstrated an ensemble approach, Dempster–Shafer theory (DST), for fusion conflicts meshed with a combination strategy for evidential weights. Their combination strategy seeks nicely for optimization on both the objective and the subjective. Classification accuracy is improved by validating against Tennessee–Eastman Process (TEP) datasets, as well as UCI machine learning datasets when compared with all other classifiers and DST-based techniques [15].

Hui et al. (2016) developed an SVM-DS model for multi-bearing fault diagnosis that utilizes Dempster–Shafer evidence theory. The model improves classification accuracy from 76% to 94 percent by resolving contradictions from individual SVM outputs and outperforms conventional fault classifiers [16]. A multi-sensor data fusion (MSDF) approach is proposed by Xiao, F. (2020), in which evidence theory is integrated with prospect theory for fusing data. In this approach, credibility measures are set, and evidence weights are adjusted, which leads to a reduction in subjective counterintuitive results within the fusion process. The experimental results highlight the MSDF’s superior effectiveness in conflict resolution and data fusion accuracy [17]. Lin, Y. et al. (2018) have built a multi-sensor model for fault diagnosis based on evidence theory with the intention of mitigating the uncertainty and conflict existing in the sensor data. Their approach was to use multiple types of sensors, such as vibration, temperature, and sound, and merge stronger evidence, which increases the diagnostic accuracy. The model proves effective in managing conflicting data for accurate fault identification [18]. Ullah, I. et al. (2021) have devised a novel data fusion framework applicable in smart environments, which is based on modified belief entropy within the Dempster–Shafer theory. This approach is useful for noisy and uncertain sensor data because of the optimization of basic probability assignments (BPAs). The method showed increased reliance and effectiveness of heterogeneous sensor fusion in decision-making systems [19]. In engineering systems, nonlinear systems fail to accurately reflect the real world due to uncontrollable variables arising from the characteristics and behavior of their components. As a result, sensor fault detection in nonlinear systems has received considerable attention owing to the complexity of maintaining the systems. Such nonlinear environments can have nonlinearity addressed by a low-compute-cost fault threshold estimation method proposed by Boucherit et al. (2017). Earlier stakeholders focused on error correction through Kalman filters [20] or neural network malfunctions when the real-time adaptive environment cannot be created. This research addresses those gaps through threshold-based methods by increasing the accuracy and adaptability of the fault detection processes. These adjustments enable improvement of the practicality of dynamic systems adaptation. In most cases, Mukhopadhyay et al. (2016) identify and highlight a necessary directional fault diagnosis modeling emphasizing evidence theory, which is largely multifaceted. Adopting this approach, the study fine-tunes reliability modeling by adding adaptive measures while maintaining a high level of accuracy in conditions that are unpredictable and non-static environments [21].

Anomaly detection contributes to the preservation of the data and operational integrity in Wireless Sensor Networks. Chirayil et al. (2019) reviewed various detection methods and pointed out the statistical and machine learning techniques while tackling the issues of resource constraints and network topology growth. Dempster–Shafer’s theory can be effectively applied to manage uncertainty emerging during sensor fusion in the context of advanced driver assistance systems (ADAS), enhancing the uncertainty management behavior and structure of fusion systems. The study concentrates on the efficient detection of faults within the limitations of dynamic and ad hoc WSNs [22]. Table 1 explains the details of existing research studies. The current methods for fire risk reduction, including AI-based models, multi-sensor fusion, and IoT-based fire detection systems, face several limitations that hinder their effectiveness in real-world applications. Many AI-powered and multi-sensor fusion models (e.g., Su et al., 2020 [10]; Wang et al., 2018 [13]) require significant computational resources, which can be a challenge for real-time fire detection, especially in large-scale environments. Additionally, Dempster–Shafer-based methods (e.g., Qiao et al., 2023 [11]; Li et al., 2017 [12]) are capable of resolving sensor conflicts but face difficulties in handling rapid fluctuations in data uncertainty, a common issue in unpredictable fire conditions. AI-driven detection models (e.g., Shadi et al., 2024; Wang et al., 2019 [15]), while improving fault diagnosis, can still generate false positives, leading to unnecessary alarms and delayed responses in dynamic fire environments. Furthermore, IoT-based systems (e.g., Ullah et al., 2021 [19]) offer remote fire monitoring but are susceptible to cybersecurity threats, network failures, and data breaches, which can undermine fire safety in critical infrastructure. These challenges highlight the need for continuous improvement in fire risk reduction and mitigation technologies to address the gaps in real-time, reliable, and secure fire detection capabilities.

### 2.2. Significant Consideration

Key considerations of this article, after performing an analysis of existing research studies, were sensor reliability, uncertain sensor data management, fire prediction, and decision-making. At the data acquisition layer [25], sensor data validation was performed using preprocessing techniques, such as normalization and anomaly detection. Faulty sensor data were addressed using sensor data pre-processing and anomaly detection [26,27], machine learning (ML) algorithms, and dynamic belief in Dempster–Shafer theory [28,29] for uncertainty quantification, ensuring reliability even with multiple faulty sensors. Challenges include real-time data processing, handling sensor conflicts, ensuring consistent data quality under dynamic fire conditions, maintaining system accuracy while managing unreliable sensors, and ensuring minimal data loss due to faulty sensors.

- Integrated Sensors Preprocessing (normal and faulty sensors): If sensor data were not pre-processed, it could lead to inaccurate, unreliable, or misleading analysis due to noise, errors, and inconsistencies. This could result in poor decision-making and reduced ML model performance. The proposed approach focused on preprocessing sensor data from multiple sensors by evaluating the sensor readings in real-time using anomaly detection to identify and filter out faulty readings [30,31]. Statistical methods should be used to assess the reliability of sensor readings. Data received from multiple types of sensors should be standardized by using normalization methods. This multi-layered pre-processing supports the identification of outliers. By this combined multi-sensor [32] pre-processing approach, clean sensor data and also faulty sensor data marked as anomaly-detected were sent to the machine learning model for further analysis. The challenges include handling real-time conflicts in sensor data and ensuring the integration of faulty sensors without compromising the overall system’s accuracy and managing sensor inconsistencies.
- Machine Learning-Based Probabilistic Model Adjustment and Sensor Reliability Enhancement for Optimized Predictions: It was recognized that sensor data would still have issues like noise, missing data, outliers, sensor drift, and other sensor-related problems. The proposed method used machine learning (ML) algorithms [33,34], which identified complex patterns and relationships within data. ML models learned and adapted to changing data over time and were designed to make predictions or classifications. The approach utilized machine learning algorithms to dynamically adjust probabilistic models based on sensor reliability and status, filtering out unreliable or faulty data. A combined weight factor, determined by the ML algorithms, was applied to each sensor’s data, resulting in precise predictions, even in the presence of unreliable or faulty sensors. Challenges include ensuring that the ML models remain robust and adaptable under changing conditions, processing large volumes of data in real-time, and making precise fire predictions despite unreliable sensor data.
- Improved Fire Prediction by Handling Sensor Data Dynamically: Relying on incomplete or faulty sensor data [35] could lead to inaccurate fire predictions. The proposed method ensured real-time adaptation to new sensor data, improving prediction accuracy. It handled conflicting sensor inputs [36] and provided timely updates, allowing the system to respond to changing conditions. This led to more reliable decision-making for fire detection. Challenges could be ensuring that the fire prediction model could adapt dynamically in real-time, handle unpredictable fire conditions, manage conflicting sensor data, and provide accurate predictions without compromising system reliability. Moreover, the model had to balance environmental changes and sensor data inconsistencies while ensuring that no critical fire risks were missed.
- Uncertainty Measurements and Decision-Making by Handling Faulty Sensors: This approach improved fire prediction accuracy by handling uncertainty and conflicting sensor data [37]. When multiple sensors provided data, the similarity and uncertainty between sensor readings and faulty sensor readings were measured to ensure reliable inputs. Using Hellinger and Deng Entropy, the Dempster–Shafer theory [38] with combined BPA helped combine multiple sources of evidence, even when data were incomplete or contradictory [39]. By combining belief and plausibility, this approach made real-time fire predictions that adapted to changing conditions, including sensor data issues. Challenges include the computational complexity of integrating multiple data sources in real-time while ensuring timely and accurate decision-making under unpredictable fire conditions was a major difficulty. Additionally, managing uncertainty in sensor data while ensuring effective and accurate fire predictions remained a significant challenge.

## 3. Hybrid Machine Learning-Based Fault-Tolerant Fire Prediction and Decision-Making

In this section, we discuss the proposed architecture overview and the methodological flow of the proposed architecture. Existing research studies’ challenges are faulty sensor data [23], efficiency, delayed response, and accuracy in fire prediction. Thus, this section discusses the proposed method for fire prediction and decision-making in the IIoT environment.

### 3.1. Proposed Architecture

**Figure 2:**
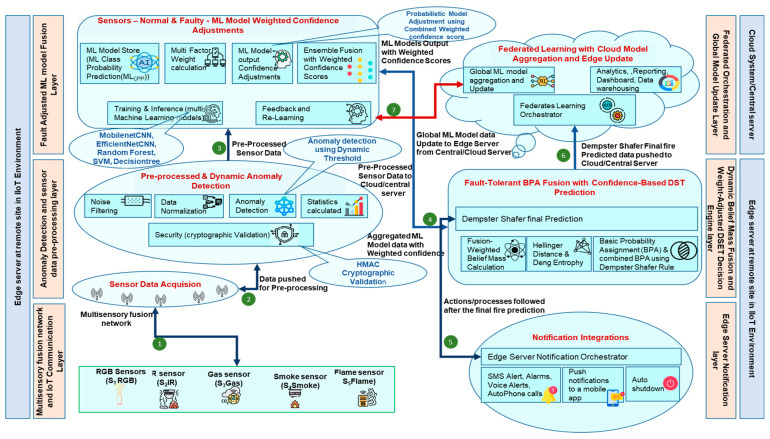
Proposed architecture for fire prediction and decision-making in IIoT environment.

An overview of the proposed method for fire prediction and decision-making in an IIoT environment is shown in Figure 2, based on core technologies that include multi-sensor fusion, sensor pre-processing, and anomaly detection, machine learning models, probabilistic model adjustment based on sensor reliability, and Dempster–Shafer evidence theory (DSET) [40]. There are multiple layers in the proposed architecture, and every layer delivers a particular function and solves specific challenges, thereby reducing the risks related to fire in the IIoT environment:

- Multi-sensory Fusion Network and IoT Communication Layer: This layer consists of IoT devices and fire sensors; multiple types of sensors are used [41]. This work addresses the limitations of relying on a single sensor type, which may lead to false positives. Research is still in progress to have reliable multi-sensor fusion [42] and to provide the prediction for fire. Fire sensors collect and transmit data using LoRa over long distances. LoRaWAN Gateway receives the data from fire sensors and forwards it to the edge server locally. This type of technology solves the challenges of network range, battery life, security, and network scalability.
- Anomaly Detection and Sensor Data Pre-Processing Layer: The greatest challenge in predictive fire analysis is obtaining accurate data, as various conditions can affect the sensor readings, making the data unreliable and unclean, or it can be a faulty sensor [43]. After data acquisition ($S_{1}DA,\;S_{2}DA,\;S_{3}DA,\;S_{4}DA,\;S_{5}DA$), this layer pre-processes the sensor data by accounting for the statuses of all sensors, including faulty ones and preprocessed {$S_{1}DP,\;S_{2}DP,\;S_{3}DP,\;S_{4}DP,\;S_{5}DP$} output given to the ML model.
- Fault-Adjusted ML Model Fusion Layer: This layer is critical, as sensors may be faulty, degraded, or affected by noise, leading to inaccurate data [44]. This is solved by deploying multiple machine-learning models specific to every sensor type. Different machine learning models, specifically MobileNetCNN (for RGB image detection), EfficientNetCNN (for IR heat detection), RandomForest (for gas classification), SVM (for smoke), and decision tree (for flame detection), are used to extract evidence from sensor data, and by integrating and adjusting the multiple sensor inputs to enhance the overall model’s reliability by filtering out faulty or irrelevant data, ensuring more accurate fire predictions {$ML_{1},\;ML_{2},\;ML_{3},\;ML_{4},\;ML_{5}$}.
- Dynamic Belief Mass Fusion and Weight-Adjusted DSET Decision Engine layer: In an IIoT oil and gas environment where we have more uncertainties, a solution is required to make decisions in environments with conflicting, incomplete, or ambiguous data, and faulty sensors. This Dempster–Shafer evidence theory [45] decision engine layer combines uncertain or imprecise information from multiple sensors and handles faulty sensor data, supporting making decisions and fire predictions related to fire ($m_{combined}(A))$ dynamically and supporting reducing risks related to fire.
- Federated Orchestration and Global Model Update Layer: This layer solves the issues of data privacy risks, data security concerns, and increased data transfer costs from local to cloud servers as sensor data are not shared with the cloud. ML models are trained locally on each remote site, and only the final model updates are sent to the central/cloud server. Global model updates are sent back to the remote sites to update their local models. This supports model updates in the remote sites with global model updates, which enhance the overall model’s reliability and provide more accurate fire prediction by reducing false alarms.
- Edge Server Notification layer: This layer integrates with devices/applications through API for sending notifications, and this plays a critical part in fire prediction by proactively taking action in the IIoT environment. This proactive approach reduces the risks of fire.

### 3.2. Technical Flow and Problem Formulation

**Figure 3:**
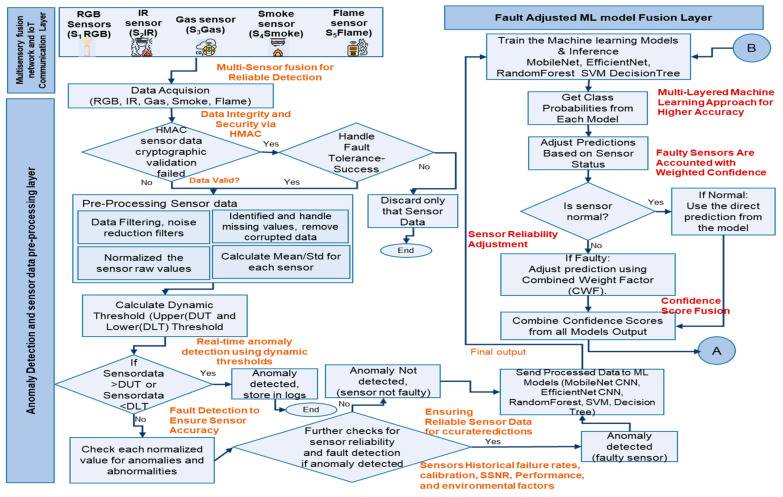
Technical flow of the proposed architecture—1.

The technical flow and problem formulation of the proposed architecture is shown in Figure 3, and this addresses the traditional fire prediction issues:

In the IIoT environment, data collections are enabled by the integration of many sensor types. Dynamic thresholds are used to manage sensor data pre-processing and aggregation, as well as anomaly detection and faulty sensor handling. Machine learning algorithms use probabilistic model adjustment to assess sensor data and filter out incorrect, irrelevant, or inconsistent inputs. Dynamic belief updates incorporate fresh evidence on a constant basis. The Dempster–Shafer evidence theory is used to predict fires, taking advantage of belief mass to account for malfunctioning sensors, thereby improving accuracy. Federated learning integrates model updates into central/cloud servers periodically, while global updates are transmitted back to remote sites and updated so every model in the remote site becomes updated by global models. The Edge Server notification module initiates API calls, sends alarms, and executes auto-shutdown actions.

**Figure 4:**
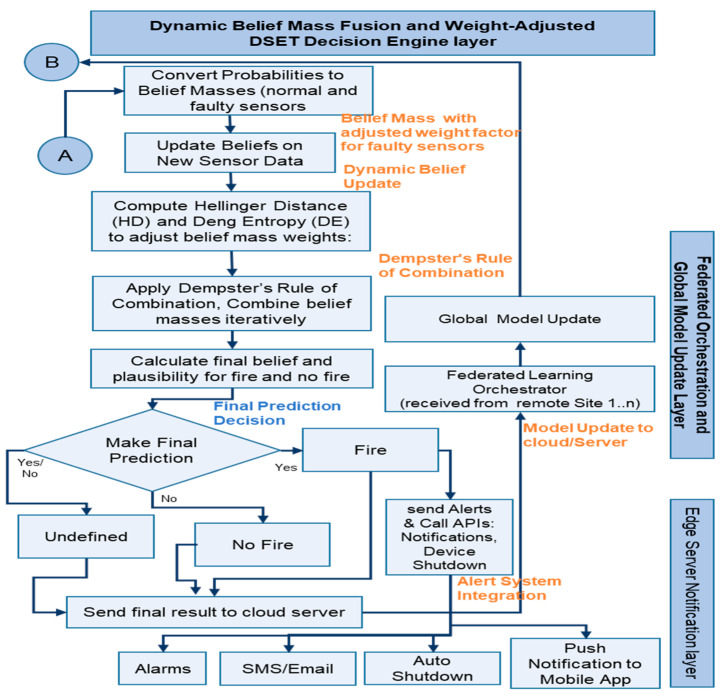
Technical flow of the proposed architecture—2.

Figure 3 and Figure 4 explain how each module technically manages a distinct functionality that takes into account faulty sensors and enhances fire prediction accuracy, thereby reducing fire risks.

Multimodal sensor integration, IoT communication layer: Multiple fire sensors ($S_{1}RGB,S_{2}IR,S_{3}Gas,S_{4}Smoke,\;and\;S_{5}Flame)$ read data from different sources, which include RGB, infrared, gas, smoke, and flame sensors. RGB data are a single data point captured from the RGB fire detection sensor camera. Infrared data are the temperature value received. The concentration of gases picked up by the sensor is shown in gas sensor data. These gases are usually carbon dioxide (CO_2_), carbon monoxide (CO), benzene (C_6_H_6_), alcohol, and other volatile organic compounds. The smoke sensor detects smoke, which includes carbon monoxide (CO), methane (CH_4_), liquefied petroleum gas (LPG), and smoke particles. The output of the flame sensor shows the intensity of a flame.

Multi-Sensor Fusion Pre-Processing and Anomaly Detection: Algorithm 1 explains the technical flow and how the sensor pre-processing pipeline works. Sensor data should not be tampered with during transmission; so, for this, a Hash-based Message Authentication Code (HMAC) cryptographic validation, SHA-256, is used. HMAC is used to validate the authenticity of sensor data by combining the data with a secret key and generating a hash.

(1) $$HSHA256(KEY,S)=H((KEY^{\prime }⊕ipad)∥H((KEY^{\prime }⊕opad)∥S))$$

where K′ is the key padded to the block size, ⊕ denotes XOR, ∥ is concatenation, S is the sensor data, iPad is inner padding, a constant hexadecimal value 0 × 36, and opad is the outer padding, a constant hexadecimal value 0 × 5C.

To handle fault tolerance, if HMAC validation fails, indicating possible data corruption, the system attempts to re-transmit the data from the sensor by sending a re-transmission request. If the data are successfully re-transmitted and pass HMAC validation, they are accepted. If re-transmission fails, the system checks for redundancy mechanisms by utilizing data from other sensors that measure the same parameters, like considering data of flame or smoke sensors when gas sensors data are missing or invalid. In cases where redundancy is not possible, the system discards only the data from the faulty sensor. Missing values in fire sensor data are identified and removed by checking for NaN or null entries. If missing values are found in any sensor reading, the corresponding data point is discarded. The result can be expressed as follows:

(2) $$Fire\;Sensor\;Data=\left.x_{i}\right|x_{i}\neq NaN$$

where NaN (not a number) is a standard way of representing missing values in datasets, so the presence of NaN is checked in the data. The formula filters out any data points where x_i_ = NaN.

Raw sensor data are normalized to ensure that sensor data with different scales are transformed to a common range of values. Multiple fire sensors, which include RGB, IR, gas, smoke, and flame sensors, have different ranges or scales, so normalization is performed to ensure sensor outputs are comparable and to avoid issues related to different scales.

(3) $$S_{i}N_{i}=\frac{\left(S_{i}-min\left(S\right)\right)}{\left(max(S)-min(S)\right)}$$

For each sensor, the mean is calculated, which is the average reading from the sensor over a period of time.

(4) $$S_{i}M=\frac{1}{n}\sum_{i=1}^{n}S_{i}N_{i}$$

For each sensor, the standard deviation is calculated, which measures how much the readings from the sensor deviate from the mean, indicating the variability of the sensor output.

(5) $$S_{i}SD=\sqrt{\frac{1}{n}\sum_{i=1}^{n}{\left(S_{i}N_{i}-S_{i}M\right)}^{2}}$$

where $S_{i}N_{i}$ represents each individual normalized reading from the sensor, and n is the number of data points received from the sensor.

Upper and lower thresholds are established for each of the five sensors, beyond which any reading is marked as an anomaly. The thresholds are calculated dynamically for each sensor and are continuously adjusted based on the latest data received from each sensor.

(6) $${DynamicUpperThreshold}_{i}=S_{i}M+\left(2\times S_{i}Std\right)$$

(7) $${DynamiclowerThreshold}_{i}=S_{i}M-\left(2\times S_{i}Std\right)$$

where i = 1 to 5 as 5 sensors are used, any reading that deviates more than 2 standard deviations from the mean will be considered anomalous.

Anomalies are detected by comparing the current sensor reading (Si) with the calculated thresholds. These data are not sent to the ML model, and it is recommended to prevent the ML model from being misled by faulty sensor values. The flagged anomalies (Si_anomaly = 1) will not affect the model directly, but they can be used for tracking faulty sensors or abnormal events in sensors and for processes like maintenance checks on them. The threshold set for calculating anomalies (Si AD) from the normalized data are based on the standard deviation of the normalized data. Normal values are close to the average, normally within a few standard deviations. Anomalous values are far from the average, and normally, they are beyond a set threshold, such as three standard deviations, which is a common threshold.

If a sensor is flagged as anomalous (S_i_AD = 1) based on standard deviation, additional checks are performed to identify sensor faults. These checks evaluate the sensor’s historical failure rate, calibration status, signal-to-noise ratio, environmental factors, and performance metrics to determine if the sensor is truly faulty. Finally, send the dictionary (S_i_DP) of all sensors containing anomaly flags, mean, standard deviation, and normalized data to the ML model.
**Algorithm 1:** Handle faulty sensors using anomaly detection during sensor-processing pipeline
**Input**:Input data from RGB sensor (S_1_ RGB), Thermal (IR) Sensor, S_2_IR, Gas Sensor S_3_Gas, Smoke sensor S_4_Smoke and Flame sensor S_5_Flame.**Output**: Normal and faulty Sensors Pre-processed output data received from Edge Computing On-Site Pre-processing server will be an input to a machine learning algorithm which are MobileNet CNN for RGB, efficientNet for IR, RandomForest for smoke, SVM for Gas and decision tree for flame.**Process:**#Acquisition of Data: Data will be continuously received from RGB, IR, Gas, Smoke and Flame sensor:S_1_DA, S_2_DA, S_3_DA, *S_4_D*A, S_5_DA#Sensor Data Pre-Processing:#Check the HMAC Cryptographic Validation, Validate each sensor’s data using cryptographic techniques: Use a shared #cryptographic key to validate the integrity of the sensor data S_1_DA, S_2_DA, S_3_DA, *S_4_D*A, S_5_DA# Handle Fault tolerance: If cryptographic validation fails, attempt re-transmission request for invalid sensor data. If re-transmission fails, check #for redundancy by using data from other sensors and replace faulty data. Discard the S_1_DA, S_2_DA, S_3_DA, *S_4_D*A*,* S_5_DA #data if no redundancy is availablefor SensorData in [S_1_DA, S_2_DA, S_3_DA, *S_4_D*A, S_5_DA]:if not validate_hmac(SensorData):ReattemptTransmission(SensorData) # Re-transmission request for invalid dataif not is_data_reliable(SensorData): # Check if redundancy mechanism can replace the failed datadiscard_data(SensorData) # Discard the data if no redundancy is available#Data Filtering, noise reduction filters applied to the RGB, IR, Gas, Smoke and Flame sensor data.#Identified and handled missing values, if missing values are found, removed the data which are affected#Normalized the sensor raw values (R_i_, G_i_, B_i_, T_i_, G_i_, S_i_, F_i_) received, S_1_N_i,_, S_2_N_i_, S_3_N_i_, S_4_N_i_, S_5_N_i._#Calculate Statistics, Mean and standard deviation#Calculate mean S_1_M, S_2_ M, S_3_M, S_4_M, S_5_M for the normalized data, For 5 sensor (R_i_, G_i_, B_i_, T_i_, G_i_, S_i_, F_i_) where i = 1 to n#Calculate standard deviation S_1_SD, S_2_SD, S_2_SD, S_3_SD, S_4_SD, S_5_SD for the normalized data for 5 sensor values ((R_i_, G_i_, B_i_, T_i_, G_i_, S_i_, F_i_) where i = 1 to n#Anomaly Detection, Implement Statistical Methods and calculate Dynamic Threshold for every sensor${DynamicUpperThreshold}_{i}=S_{i}\;M+\left(2\;*\;S_{i}Std\right)$${DynamiclowerThreshold}_{i}=S_{i}\;M-\left(2\;*\;S_{i}Std\right)$#where i = 1 to 5 as 5 sensors are used, any reading that deviates more than 2 standard deviations from the mean #will be considered anomalous.if S_i_ > DynamicUpperThreshold_i_ or S_i_ < DynamicLowerThreshold_i_, S_i__anomaly = 1 for anomalies, 0 for normal#record the anomaly detected as this may be from faulty sensor. This find whether the sensor’s reading is #anomalous based on raw, untransformed data.#Check each normalized value for anomalies and abnormalitiesif (S_i_N_i_) > 3, set the S_i_ AD = 1 else 0 where i = 1 to 5 sensors, #anomaly flag = 1#Further checks for sensor reliability and fault detection if anomaly detectedif S_i_AD[i] == 1# If sensor is flagged as anomalous, further checks are needed# Check if the sensor’s historical failure rate (FR) exceeds a thresholdif historicalfailurerate[i] > failurethreshold, S_i_AD[i] = 1 # Confirm the sensor as faulty# Check if the sensor’s calibration issue is out of acceptable rangeelif abs(S_i_N_i_[i]—expectedcalibration[i]) > calibrationthreshold,S_i_AD[i] = 1 # Confirm the sensor as faulty# Check the sensor’s signal-to-noise ratio (SSNR) if it’s below acceptable levelelif SSNR[i] < ssnrthreshold, S_i_AD[i] = 1 # Confirm the sensor as faulty# Check if the sensor’s performance metrics (PSM) show a degradation (e.g., low response)elif sensorperformance[i] < performancethreshold, S_i_AD[i] = 1 # Confirm the sensor as faulty# Check environmental impact factors (EIF), like temperature or humidity affecting sensorelif environmentalfactors[i] > environmentalthreshold, S_i_AD[i] = 1 # Confirm the sensor as faultyelseS_i_AD[i] = 0-- # If none of the checks indicate a fault, set anomaly flag to 0(normal) where i = 1 to 5 types of sensors#Send the transformed data to the machine learning algorithm Mobile Net CNN, Efficient Net CNN, Random Forest, SVM and decision tree.S_1_ DP = {“Anomaly detected”: S_1_AD, “mean”: S_1_M, “standard deviation”: S_1_SD, “normalized_data”: S_1_N_i,_ Auxiliary_data}S_2_ DP = {“Anomaly detected”: S_2_AD, “mean”: S_2_M, “standard deviation”: S_2_SD, “normalized_data”: S_2_N_i_, Auxiliary_data}S_3_ DP = {“Anomaly detected”: S_3_AD, “mean”: S_3_M, “standard deviation”: S_3_SD, “normalized_data”: S_3_N_i_, Auxiliary_data}S_4_ DP = {“Anomaly detected”: S_4_AD, “mean”: S_4_M, “standard deviation”: S_4_SD, “normalized_data”: S_4_N_i,_ Auxiliary_data}S_5_ DP = {“Anomaly detected”: S_5_AD, “mean”: S_5_M, “standard deviation”: S_5_SD, “normalized_data”: S_5_N_i_, Auxiliary_data}END

Machine learning model module with Probabilistic model adjustment: Algorithm 2 explains the technical flow of the machine learning models, including adjusted predictions for faulty sensors, dynamic belief mass, and Dempster–Shafer evidence theory, which considers both normal and faulty sensors during fire prediction and addresses existing research gaps.

Once the data are pre-processed, they are sent to the respective machine-learning models for analysis and fire prediction. Each model monitors specific sensor readings to identify the presence of fire or its related anomalies and further assists in fire prediction and detection. Furthermore, the models implement a fault-handling mechanism where the model output is adjusted, resolving issues caused by faulty sensors or missing data, thus maintaining active and accurate fire monitoring.

The normalized data (S_1_N_i_) received from RGB sensors is handled by the MobileNet CNN, which uses the mean (S_1_M) and standard deviation (S_1_SD) to provide an adjusted model output that handles faulty sensors. The EfficientNet CNN can identify temperature increases using pre-processed IR sensor data (S_2_N_i_) due to its advanced ability to analyze temperature patterns with the mean (S_2_M) and standard deviation (S_2_SD). It enables reliable fire detection by identifying heat-related anomalies or measurements. If there is a sensor drift or bias, EfficientNet can handle sensor-related issues and adjust the model output accordingly.

Random Forest is supplied with normalized gas sensor data (S_3_N_i_) along with mean (S_3_M) and standard deviation (S_3_SD) to provide a probabilistically adjusted model output. It establishes multiple decision trees to identify gas levels. The model has strong resistance to sensor faults as it can handle missing or corrupted data by leveraging other decision trees.

Support vector machines (SVMs) employ normalized smoke sensor data (S4Ni) along with the mean (S4M) and standard deviation (S4SD) to identify smoke level patterns. It differentiates air from smoke, categorizes smoke levels, and monitors its fluctuations while considering anomalies and faulty sensor data in its model output. If there is a sensor fault, SVM can recognize sensor faults, outliers, and abnormal data patterns.

Decision trees process normalized flame sensor data (S_5_N_i_) along with the mean (S_5_M) and standard deviation (S_5_SD) to assess flame intensity and variability. They build decision rules to detect flame presence and intensity, providing both a binary classification and a flame intensity score. Decision trees are robust to missing or faulty data, as they rely on available features for accurate predictions and provide a model output.

Model Output (Prediction Probabilities): Each machine learning model generates prediction probabilities indicating either “fire” or “no fire” based on the pre-processed sensor data. These models do not detect faulty sensors directly, and this is why sensor fault detection is performed in the sensor data pre-processing module.

Probabilistic Adjustment $({m\left(fire\right)}_{K},{m\left(nofire\right)}_{K}$) on model output is performed based on sensor reliability. Once a faulty sensor is identified, the model’s predictions are adjusted using a Combined Weight Factor (CWF). The CWF is a factor calculated as below:

(8) CombinedWeightfactor(CWF)=PWC×FR+PWC×CS+PWC×SSNR+PWC×PSM+PWC×EIF

where PWC is percentage of weight (%) for calculating combined weight factor, FR is failure rate based on historical data, CS is calibration of sensors, SSNR is the sensor’s signal-to-noise ratio, PSM is the performance of sensor by metrics, and EIF is environmentally impacted factors.

The purpose of this adjustment is to account for the sensor’s reliability. If the sensor is faulty (due to calibration drift, high noise, or other issues), the weight of its fire prediction is reduced. This ensures that unreliable sensor data does not significantly influence the final fire prediction in the IoT-based oil and gas industrial environment. The total confidence (TotlConf) score is calculated by summing the individual model outputs for both fire and no-fire probabilities across all sensors. The formula aggregates the confidence scores for each model (both normal and faulty) using weighted contributions, where each model’s confidence is adjusted based on sensor reliability, ensuring that the final decision reflects the overall system’s confidence in fire detection.

(9) $$TotlConf=\sum_{K=1}^{n}{m(fire)}_{K}+\sum_{K=1}^{n}{m(nofire)}_{K}$$

Dempster–Shafer Evidence Theory [46]: The belief masses for fire, no fire, and the entire hypothesis are calculated by converting the class probabilities (m_i_(fire), m_i_(no fire), and mi(Θ)) from each machine learning model into weighted belief masses. For fire and no fire, the belief masses are obtained by multiplying the class probabilities with the corresponding weight factors for each sensor and then normalizing the sum by the total confidence. Sensors flagged as faulty have their weight factors reduced (to less than 1), decreasing their influence on the final belief mass. The system calculates the belief mass for the hypothesis (m(Θ)) by taking 1 and subtracting the difference between values of fire and no fire belief masses making sure the total probability distribution adds up to 1. This approach ensures that the faulty sensors have less impact on the belief mass calculation so that the weight factor is applied based on sensor status which can be normal or faulty. When the system puts together the results from all models with these adjusted weights, it increases the accuracy and dependability of its fire detection and predictions.

Belief mass for fire

(10) $$m\left(fire\right)=\sum_{K=1}^{n}\frac{P_{k}\left(fire\right|S_{K})\times {weightfactor}_{K}\}}{TotlConf}$$

Belief mass for no fire

(11) $$m\left(no\;fire\right)=\sum_{K=1}^{n}\frac{P_{k}\left(no\;fire\right|S_{K})\times {weightfactor}_{K}\}}{TotlConf}$$

where normal sensors’ weight factor will be configured as 1 and faulty sensors’ weight factor will be set less than 1.

Belief mass for the entire hypothesis

(12) $$m\left(\Theta \right)=1-(m\left(fire\right)-m\left(no\;fire\right))$$

When the belief mass $M_{New}\left(fire\right))$ is dynamically updated, it ensures that the model remains very responsive to real-time data received from ML models. It also considers past data information, which results in more accurate fire detection. The weighting factor α helps control how much the past belief influences the current data received. To have a balance between historical data and current observation, 0.5 for α is considered. This will ensure that historical and current data do not impact the model. In an oil and gas IIoT environment, sensor data fluctuates because of environmental conditions, this proposed approach supports reliable fire prediction.

(13) $$M_{New}(fire)=\alpha \cdot M_{Previous}(fire)+(1-\alpha )\cdot P_{k}(fire|S_{k})$$

where α is a weighting factor between 0 and 1. This weighting factor α supports in controlling how much the past belief has influence when compared to current data received. For this analysis, 0.5 is used. The Hellinger Distance (HD)($HD\left(m_{i},m_{j}\right))$ and Deng Entropy (DE) (DE(mi)) are used to resolve the problem of inconsistent or uncertain data by assessing the similarity and uncertainty between various data received. HD helps measure how much two belief masses (model outputs) differ, ensuring that the system gives more weight to similar, reliable sensor data and reduces the impact of conflicting predictions. DE quantifies the uncertainty in each sensor’s belief mass, helping to identify unreliable or less confident sensor outputs, thereby improving overall decision-making accuracy in fire detection. These measures ensure that sensor data fusion is more robust and trustworthy. It is assumed that sensor issues occur frequently, so α was set to 0.4 and β to 0.6. A high alpha (α) focuses more on the similarity between sensors, ensuring that sensors providing similar outputs are trusted more. A high beta (β) focuses more on the uncertainty of the sensor outputs, which is useful when sensors are unreliable or prone to errors. Integrating both HD and DE into the sensor fusion algorithm allows the system to handle conflicting predictions while prioritizing more reliable sensors, leading to better overall fire detection performance. The algorithm applies Dempster’s Rule of Combination (m_Combined(A)) to iteratively combine belief masses from multiple machine learning models based on sensor inputs. It computes the combined belief mass for each class by merging the evidence from each model while resolving conflicting information. After applying the rule, the belief (Belief(A)) and plausibility (Plausibility(A)) values for fire and no fire are calculated.

The final fire predictions are made by comparing the belief values against predefined thresholds. If the belief in fire or no fire exceeds the threshold, the respective class is predicted. Otherwise, the fire prediction remains undefined or uncertain. This ensures more reliable and accurate fire predictions and addresses the problem of sensor uncertainty and conflicts, ultimately mitigating fire risks in IIoT environments.
**Algorithm 2:** Adjusted prediction for faulty sensors and dynamic belief mass for final fire prediction
**Input**: Multi Sensor fusion pre-processed data {S_1_DP, S_2_DP, S_3_DP, S_4_DP, S_5_DP}**Output:** the final prediction of fire based on belief and plausibility values considering normal and faulty sensors**Process**:#Train machine learning Model, MobileNet on RGB pre-processed sensor Data, EfficientNet on IR pre-processed #sensor #Data, RandomForest on Smoke pre-processed sensor Data, SVM on Gas pre-processed sensor#Data, DecisionTree on Flame pre-processed sensor Data#Get Class probabilities prediction from each Model for 5 different multi sensor for fire and NoFire.      ML_1_ = MobileNet(S_1_DP)      ML_2_ = EfficientNet(S_2_DP)      ML_3_ = Randomforest(S_3_DP)      ML_4_ = SVM(S_4_DP)       ML_5_ = DecisionTree (S_5_DP) #adjusted prediction based on sensor status and model output is calculated.CombinedWeightfactor(CWF)=PWC×FR+PWC×CS+PWC×SSNR+PWC×PSM+PWC×EIF#Percentage of weight (%) for calculating combined weight factor(PWC), Failure rate based on #historical data(FR), Calibration of sensors(CS), Sensor signal to Noise Ratio(SSNR), #performance of sensor by metrics(PSM), environmental impacted factors(EIF) #Probabilistic model adjustment based on sensor reliability. each Model, gives probabilities for #fire and no fire:If S_i_AD = 0 then--# If Si(sensor) is normal,$\left({m(fire)}_{K}=P_{k}\left(fire\right|S_{i}\right)$${m\left(no\;fire\right)}_{K}=P_{k}\left(no\;fire\right|S_{i})$Else --# if Si(Sensor) is faulty${m(no\;fire)}_{K}=P_{k}\left(no\;fire\right|S_{i})xCWF$${m(fire)}_{K}=P_{k}\left(fire\right|S_{i})xCWF$#where CWF< 1 is to reduce the confidence from faulty sensors.**#** Si   where i ranges from 1 to 5 of sensors S_1_, S_2_ … S_5_ which are RGB, IR, Gas, Smoke and Flame#sensors and PK is a particular machine learning model respective to that sensor which are ML_1_, ML_2_, ML_3_, #ML_4_, ML_5_ from where probability is derived for fire and no fire. #Combine the confidence score from all models (both normal and faulty sensors), Ensemble Fusion with Weighted #Confidence Scores$TotlConf=\sum_{K=1}^{n}{m(fire)}_{K}+\sum_{K=1}^{n}{m(no\;fire)}_{K}$#Convert the class probabilities mi(fire), mi(no fire),mi(Θ)from each machine learning models into belief masses. #Fusion-Weighted Belief Mass Calculation#Belief mass for fire$m\left(fire\right)=\sum_{K=1}^{n}\frac{P_{k}\left(fire\right|S_{K})X{weightfactor}_{K}\}}{TotlConf}$#Belief mass for no fire$m\left(no\;fire\right)=\sum_{K=1}^{n}\frac{P_{k}\left(no\;fire\right|S_{K})X{weightfactor}_{K}\}}{TotlConf}$#Where for Normal sensors weight factor will be set to 1, while faulty sensors factor is set less than 1 to reduce #their impact.#Belief mass for entire hypothesis$m\left(\Theta \right)=1-(m\left(fire\right)-m\left(no\;fire\right))$#Dynamically Belief is calculated, the new belief is based on the latest sensor readings.$M_{New}(fire)=\alpha \cdot M_{Previous}(fire)+(1-\alpha )\cdot P_{k}(fire|S_{k})$      #Where α is a weighting factor between 0 and 1 that determines how much influence the previous       #belief mass has compared to the new evidence from the current sensor reading. 0.5 is used for       #Balanced approach as the IoT environment is assumed to be stable environments#calculate the Hellinger Distance H(m_i_,m_j_)  between each pair of  belief masses $HD\left(m_{i},m_{j}\right)=\frac{1}{\sqrt{2}}\sqrt{\sum_{k=1}^{n}{\left(\sqrt{m_{i}\left(k\right)}-\sqrt{m_{j}\left(k\right)}\right)}^{2}}$#Deng Entropy DE(mi) is calculated for each machine learning model of sensors, the belief masses are adjusted   $DE(mi)=-\sum_{\left\{k=1\right\}}^{\left\{n\right\}}m_{i}\left(k\right)log(m_{i}\left(k\right))$   $w_{i}=\frac{1}{1+\alpha \cdot HD\left(m_{i},m_{j}\right)+\beta \cdot DE(m_{i})}$ #α controls how much weight the Hellinger Distance has in adjusting the belief mass. #β controls how much weight the Deng Entropy has in adjusting belief mass.
8.#Apply Dempster’s Rule of Combination iteratively for all pairs of belief masses       $m_{Combined}(A)=\frac{\sum_{\left\{B\cap C=A\right\}}w_{1}m_{1}\left(B\right).w_{2}m_{2}(C)}{(1-\sum_{B\cap C=\emptyset }w_{1}m_{1}\left(B\right){.w}_{2}m_{2}(C))}$
9.#After combining the belief masses, compute the belief and plausibility for each class, The final belief mass mcombined #provides the combined evidence,      $Belief\left(A\right)=\sum_{B\cap C=A}m_{combined}(B)$      $Plausibility(A)=\sum_{B\cap A\neq \emptyset }m_{combined}(B)$10.#Dempster’s Rule is applied to combine belief masses mcombined(A) from all models, Repeated this combination #iteratively for all pairs of machine learning models of every multi sensors inputs11.#After combining the BPAs, calculated the belief and plausibility for each class Belief(A) and Plausibility(A)12.Determined the final prediction of fire based on belief and plausibility values
The final prediction was made based on the calculated belief and plausibility values:                     $If\;Belief\left(fire\right)>threshold,Predict=“fire”$                     If Belief(no fire)>threshold,Predict=“no fire.”
Otherwise, predict “undefined” or “uncertain”.
13.#When fire predicted, API is called for SMS(POST/alert/sms), email (POST/alert/email),#Push notification (POST/alert/push), and also API called for auto showdown (POST/alert/shutdown) of devices.14.#Send the final prediction to cloud/central server by calling the API (POST/models/update). Global Update #centralized model training received from all the remote sites through secured and authenticated API. Periodically #this global model will be updated back in the machine learning model in every remote sites edge server using API #(POST/models/update-global).
END

When a fire is detected, the edge server triggers multiple alert mechanisms via API’s for timely response and damage prevention. These include sending SMS notifications (POST/alert/sms) and email alerts (POST/alert/email) to relevant personnel, activating auto-shutdown actions (POST/alert/shutdown) to stop critical equipment, and pushing real-time push notifications (POST/alert/push) to connected IoT devices. These actions ensure safety and prompt intervention for taking action. In function 7, which is Federated Learning, local model updates (parameters) are periodically transmitted from edge servers to a central server via a secure API (POST/models/update). The central server aggregates these updates using techniques such as Federated Averaging (FedAvg) to form a global model by ensuring model aggregation and updates from multiple remote sites having multiple types of sensors. The updated global model is then pushed back to edge devices using POST/models/update-global, maintaining synchronization across remote sites. To ensure secure transmission, both updates are encrypted, and authentication mechanisms are implemented. The model’s performance is continuously monitored. The global model’s update frequency is optimized based on network bandwidth, computational resources, and sensor data inconsistencies to balance accuracy and efficiency.

## 4. Results

This section discusses the results, performance, and evaluation of the proposed architecture for fault-tolerant sensor data fusion and anomaly detection for fire risk mitigation in the IIoT environment. We predicted fire prediction in the IIoT environment using multiple sensor fusion and handling sensor inconsistencies or faulty sensors during sensor pre-processing in the hybrid machine learning algorithm by adjusting the predicted model outputs and by updating and adjusting the DSET belief mass values considering the faulty sensors. The research focuses on fire prediction using sensor fusion and preprocessing, considering sensor inconsistencies and faulty sensors. In the design, the output data are given to multiple ML models, and the ML model outputs are further refined, and probabilistic adjustments are performed based on sensor data status. We feed these data to the Dempster–Shafer belief mass, which then calculates the Fusion-Weighted Belief Mass. Dempster’s rule of combination is iteratively applied for all pairs of belief masses. After combining the belief masses, the belief and plausibility for each class were calculated, and the final prediction of fire was performed based on belief and plausibility values. We evaluated the performance and efficiency of the proposed approach using metrics that enhanced performance visibility. We evaluated the proposed approach by deploying the solution on an edge server, having Raspberry Pi and Python 3.11.4 used for scripting and data processing. As machine learning models were used, TensorFlow Lite was used on edge devices. Sensor quality and preprocessing significantly impact the DSET outcomes. We used data from online sources and also simulated data. Perfect sensor data cannot be guaranteed in the IIoT oil and gas environment, and hence the proposed approach handles situations when inconsistent or faulty sensor data are received. Table 2 explains the metrics details of the proposed method. It was noted that when no anomalies were detected, the proposed approach achieved high precision, recall, F1-score, and accuracy.

**Figure 5:**
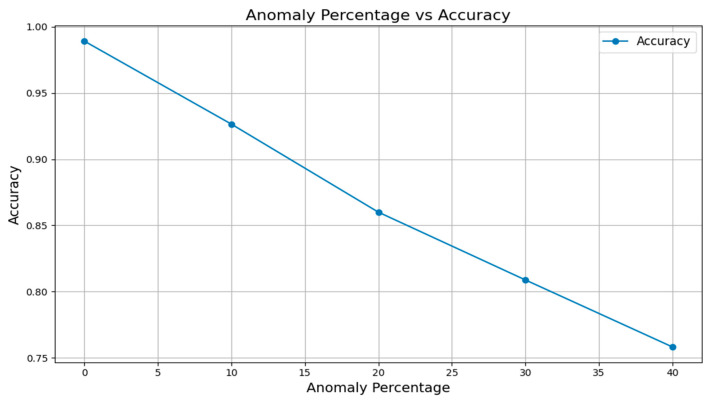
Anomaly percentage vs. Accuracy.

This evidence indicates that the fire predictions were reliable under normal conditions. As the anomaly value increased to 10%, 20%, 30%, and 40%, all metrics showed a drop, which demonstrates the system’s impact in managing increased uncertainty and sensor data inaccuracies. But still, the proposed approach provided higher precision and accuracy when it was experimented with the anomaly of 40%. Even when no sensors failed, the anomaly impacted the fire prediction, and with a 40% anomaly, the accuracy of fire prediction in the oil and gas IIoT environment was 75.8% and precision was 84.9%. Figure 5 shows the proposed approach and how it provides accuracy even under multiple conditions of anomaly percentage.

**Figure 6:**
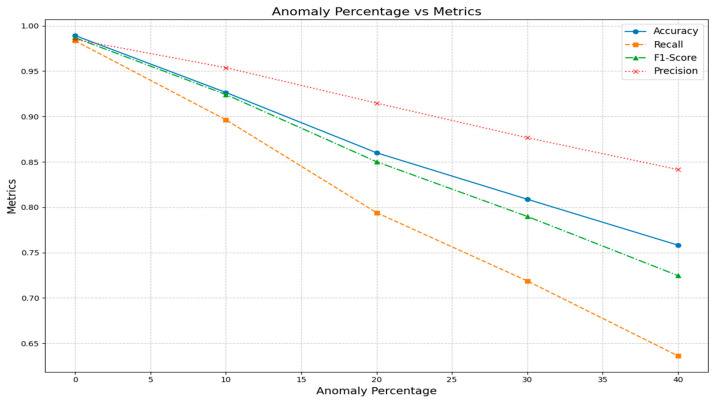
Anomaly vs. accuracy, recall, F1-score, and precision.

The proposed approach takes into account inconsistent or faulty sensor data and anomalies. The final fire prediction data, obtained from multiple ML models, DSET, and the performance metrics, were depicted below. Anomalies of 10%, 20%, 30%, and 40% were introduced, and the corresponding metrics were evaluated, as detailed in Figure 6. Even though anomalies were considered during fire prediction, it is still mandatory to address the anomaly data to achieve high fire prediction accuracy. To verify the impact and performance of the proposed approach, sensors were failed, and their impact on fire prediction was researched. When one sensor failed, the proposed approach performed well. Precision, recall, F1-score, and accuracy were high.

Table 3 shows the performance metrics with a simulation of the failing sensor. When four sensors failed, precision was 88%, and accuracy was 67.7% in predicting fire with the availability of limited sensors and data.

**Figure 7:**
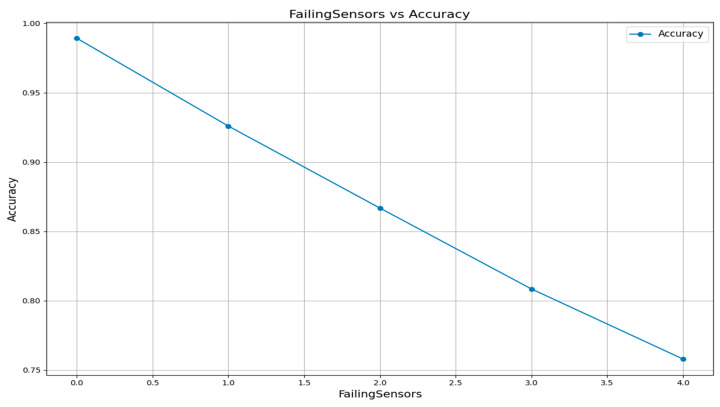
Failed sensors vs. accuracy for fire prediction in IIoT environment.

This sensor-related analysis reveals its impact on the reliability of the fire prediction. The accuracy drops when the failing sensor increases. Figure 7 shows it is mandatory to act on the failed sensors so fire prediction accuracy will be high.

**Figure 8:**
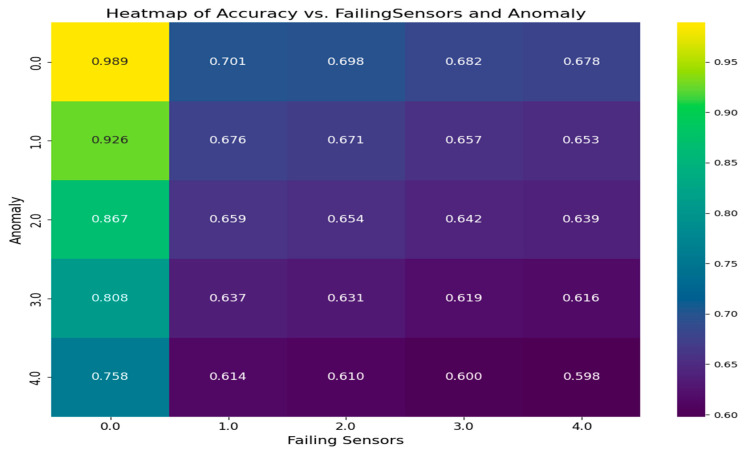
Heat map of accuracy vs. failing sensors and anomaly.

Figure 8 explains the heat map of the accuracy of fire prediction by comparing it against failed sensors and anomalies. The *X*-axis represents the impact of failed sensors, ranging from no failure to all failures. The *Y*-axis represents the anomaly, which is the deviation of sensor normal data; this will range from no anomalies to 40% anomalies. Low anomaly and no sensor heat map failure result in accurate predictions because the sensor data are normal and there are no failures. In the case of low anomaly and sensor failures in the heat map, where sensor failures are introduced but the anomaly level is low, the algorithm is still able to maintain accuracy, as sensor faults are handled during sensor pre-processing using a dynamic threshold. In the case of high anomaly and no sensor failure, when anomalies are introduced, challenges may arise, but the sensors still function properly. The proposed method and algorithm for handling anomalies contribute to achieving better accuracy. However, in high anomaly and sensor failure scenarios, the accuracy is the lowest, as both anomalous data and faulty sensors introduce complexities for the algorithm. Despite this, as sensor faults and anomalies are handled, the accuracy remains relatively higher even in this scenario. Unlike other studies that assume sensors are always reliable, this proposed model adjusts confidence scores based on how healthy sensors are in real-time, so performance stays stable even when there are sensor issues.

Conventional anomaly detection approaches rely on binary thresholds, limiting their ability to handle complex variations in sensor data. In contrast, these methods integrate multiple sensor performance metrics and environmental impact factors, providing a more refined and adaptive fault assessment. Additionally, while many prior studies depend on single classifiers, these approaches employ an ensemble of ML models (MobileNet, EfficientNet, RandomForest, SVM, DecisionTree), enabling sensor-specific learning and improving robustness across diverse environments. The results further validate these model’s reliability, demonstrating that even with 40% anomalous sensor data, it maintains high fire prediction accuracy through redundant sensor cross-validation, weighted confidence adjustments, and dynamic probabilistic fusion. Moreover, the model was highly scalable, processing data from multiple heterogeneous sensors across various industrial settings, including oil and gas sectors (upstream, midstream, and downstream). Unlike traditional models that struggle with large-scale data handling, this system ensures continuous learning through global model updates via API, allowing for real-time adaptability. These results confirm that the proposed approach was both scalable and accurate, effectively mitigating the impact of anomalies and ensuring reliable fire detection in dynamic environments, thereby reducing and mitigating fire risks in IIoT environments.

## 5. Discussion

The proposed architecture parameters were compared with the existing studies. This section discusses fire prediction and decision-making in an IIoT environment, considering the critical issue related to faulty sensors. We also compared it with the existing research work. Existing research work is shown in Table 4. In this analysis, we have used very critical parameters and showed how the proposed architecture and approach fulfilled all parameters. Fire sensors can experience a range of faulty issues, such as sensitivity problems, power failures, and calibration errors, leading to false alarms or missed fire detection. Sensor aging, mechanical damage, software issues, and communication failures can create sensor issues and increase the risk of undetected fires. Compared to other research studies, which are dependent on fixed thresholds or periodic updates to predict fire, the proposed approach works on continuous adaptation to the real-time updating of sensor data, considering all sensor data issues mentioned above, and this improved the system to respond with higher performance in dynamic oil and gas IIoT environments. The proposed approach analysis was based on sensor fusion, anomaly detection, machine learning model prediction, real-time adaptation to sensor faults, uncertainty handling, Fusion-Weighted Belief Mass, dynamic belief update, and data quality and integrity. These methods support the reduction in fire risks in IIoT environments.

- Sensor Fusion: The proposed architecture combines inputs from multiple unique types of sensors, each of which detects different types of fire. This ensures that multiple sensors reduce false positives or missed fire predictions [47]. As multiple unique types of sensors are used, early detection is possible as gas or smoke can be detected before the actual flame of fire or heat. This will result in faster response times and results in reducing the risks of fire.
- Adaptive Anomaly Detection for Fire Detection: As these sensors will be in an IIoT environment in remote sites, sensor data can be impacted because of various factors like temperature, location, etc. Anomaly detection with dynamic threshold support is also used in real-time to assist in the early prediction of fires. Anomalies found across multiple sensor data and abnormal sensor data are flagged. This procedure also reduces false predictions of fire as it differentiates between random sensor-related noise data and actual anomalies, which supports reducing fire risks.

**Table 4:** A comparative study of the proposed approach and existing research.

Existing Approach	Sensor Fusion	Adaptive Anomaly Detection	Multi ML Models Adjusted Prediction	Real-Time Adaptation to Sensor Faults	Uncertainty Handling	Fusion-Weighted Belief Mass	Decision-Making in Evolving Environments	Data Quality and Integrity
Sharma, M et al., 2022 [48]	Yes	No	No	No	Yes	No	No	Yes
Sung, S et al., 2024 [49]	Yes	Yes	No	No	No	No	No	Yes
Çavdar, T et al., 2022 [50]	Yes	No	No	No	Yes	No	No	Yes
Sharma, M et al., 2023 [51]	Yes	No	No	No	Yes	No	No	Yes
Ghosh, N et al., 2020 [24]	Yes	Yes	No	No	Yes	No	No	Yes
**Proposed Approach**	Yes	Yes	Yes	Yes	Yes	Yes	Yes	Yes

- Multi-ML Models Adjusted Prediction Based on Faulty Sensors: Machine learning models process the pre-processed sensor’s data. The combined weight factor adjusts the individual model predictions based on sensor status, either normal or faulty. This improves reliability, as this ensures that the faulty sensor data do not impact the fire prediction, and the proposed approach is supported to rely more on sensors that are functional and less on faulty sensors, which prevents false fire prediction and resulted in reducing the fire risks in such a complex IIoT environment.
- Real-Time Adaptation to sensor faults: In real-time, during sensor preprocessing, sensor faults or inconsistent sensor data are dealt with. Machine learning outputs are changed based on the status of faulty sensors, and the Dempster–Shafer Fusion-Weighted Belief Mass combines data and takes sensor status into account. This approach avoids inaccurate fire predictions caused by unreliable sensor data. Data are aggregated from various sources, and this provides more robust and accurate fire prediction as sensor faults are considered. The proposed approach ensures that faulty sensors never impact the final decision, and this improves the overall performance and accuracy of the fire detection system we proposed. This further supports in reducing the fire risks in the IIoT environment.
- Uncertainty Handling: The Dempster–Shafer theory approach handles uncertain, inconsistent, or faulty sensor data. This method helps in making decisions about fire predictions even when there is incomplete or conflicting sensor data, thereby supporting the reduction in fire risk in oil and gas industrial environments [52].
- Fusion-Weighted Belief Mass: Data from several machine learning models were combined, and inconsistent or unclear data were handled by giving faulty sensors extra weight [53,54]. This approach enhances decision-making accuracy by accounting for sensor failures and utilizing fire-related sensor data from critical oil and gas IIoT environments, thereby reducing the risks of fire.
- Stable Decision-Making In Evolving Environments: This attribute is critical for decision-making, as evidence was combined from multiple ML model outputs. Dynamic belief updates in the proposed approach were updated based on historical beliefs and new sensor data, which support adapting to changing environmental conditions in the IIoT environment. As current sensor readings were considered, this helps in reducing the risk of fire in a complex dynamic changing environment.
- Data Quality and Integrity: When a sensor fails, the proposed approach ensures data quality and integrity by employing sensor data fusion where readings from multiple sensors are combined to provide more accurate results. Multiple algorithms handle faulty sensor readings. Compared to other research studies, the proposed approach dynamically receives data from multiple fire-related sensors in real time, and it supports the reliability and accuracy of fire prediction even under sensor failures.

## 6. Conclusions

In this research article, a fire prediction system was designed that handled sensor faults in an IIoT environment. Sensors in the IIoT environment were impacted by multiple conditions such as environmental interference, aging, or sensor hardware issues, which resulted in sensor data inconsistencies. During the research, it was observed that faulty sensors introduced noise or missing data, leading to false alarms or missed fire events. Multiple types of sensors were deployed, and each sensor’s reliability was critical for detecting fire accurately. The issue of faulty sensors was addressed in multiple ways; a dynamic threshold was implemented during the preprocessing phase. This approach adjusted the thresholds of sensor readings based on the real-time data received. For example, when a sensor’s fault was detected, its threshold was adjusted dynamically by reducing its influence. This adjustment supported the maintenance of the system’s stability and prevented faulty sensor data from impacting the overall fire detection process. The machine learning models were trained on pre-processed sensor data, and faulty sensors were considered in every model output. By adjusting the ML model’s output based on the sensors’ statuses, the impact of faulty sensor readings was handled effectively. It was also found that the performance of the machine learning models was impacted when multiple sensors failed simultaneously. A weight factor was used based on sensor status and anomalies, and the belief masses in the Dempster–Shafer evidence theory (DSET) were adjusted; this approach arrived at accurate fire predictions. DSET provided a valuable solution for handling uncertainty and belief fusion. By dynamically adjusting belief masses based on sensor performance, the system gave more weight to normal sensors while compensating for the errors introduced by faulty sensors. The ability to update belief masses in real time allowed for continuous adaptation, which was crucial for maintaining fire detection accuracy in dynamic and uncertain environmental conditions in the oil and gas IIoT environments. Future research aimed to explore other AI models that could be trained to automatically calibrate sensors and handle faulty sensors in a resource-constrained environment. Additional environmental factors, such as wind speed and humidity, need to be considered for exploration, and new sensors/data sources can be integrated into future research.

## Figures and Tables

**Table 1:** Details of existing research studies.

Author(s) and Year	Objective/Research Problem	Methodology	Key Findings	Technology
Su, Z et al., (2020) [10]	Develop a robust fault diagnosis model combining advanced feature extraction, optimized classification methods, and multi-sensor data fusion for enhanced performance.	Singular value manifold features, support vector machines (SVMs)	Improves diagnostic accuracy and robustness in industrial fault diagnosis scenarios	Singular Value Decomposition (SVD), optimized SVM algorithms, and multi-sensor data fusion techniques.
Qiao, S et al., (2023) [11]	Enhance multi-sensor data fusion reliability for unmanned surface vehicles using belief divergence.	Fuzzy Dempster–Shafer evidence theory method with belief divergence adjustment.	Improved fusion accuracy and robustness.	Multi-sensor data fusion, unmanned surface vehicles, fuzzy Dempster–Shafer framework.
Li, S et al., (2017) [12]	Improve fault diagnosis of bearings by enhancing the fusion of deep learning outputs.	Deep convolutional neural networks with Dempster–Shafer evidence fusion.	Enhanced fault diagnosis accuracy.	Deep learning, bearing fault diagnosis, Dempster–Shafer evidence fusion.
Wang, Z et al., (2018) [13]	Develop a more effective multi-sensor data fusion approach for fault diagnosis.	Multi-sensor data fusion algorithm integrating reliability assessment.	Better fault diagnosis reliability.	Multi-sensor data fusion, fault diagnosis systems.
Wang, Z et al., (2019) [15]	Enhance fault recognition accuracy using advanced classification techniques.	Ensemble classifier integrated with Dempster–Shafer theory.	Improved fault recognition performance.	Fault recognition, ensemble classifier, Dempster–Shafer theory.
Hui, K. et al., (2016) [16]	Diagnose multiple bearing faults using advanced evidence fusion techniques.	Dempster–Shafer evidence theory for fault analysis.	Accurate multi-bearing fault diagnosis.	Bearing fault diagnosis, Dempster–Shafer evidence theory.
Xiao, F. (2020) [17]	Improve multi-sensor data fusion accuracy through enhanced evidence combination.	Evidence combination using prospect theory and Dempster–Shafer framework.	Enhanced fusion robustness and precision.	Multi-sensor data fusion, prospect theory, Dempster–Shafer evidence theory.
Lin, Y et al., (2018) [18]	Develop a fault diagnosis model integrating data from multiple sensors.	Evidence-theory-based fault diagnosis modeling.	Reliable multi-sensor fault diagnosis	Multi-sensor data fusion, evidence theory.
Ullah, I et al., (2021) [19]	Improve data fusion in smart environments by addressing belief uncertainty.	Modified belief entropy in Dempster–Shafer theory.	Enhanced fusion accuracy and reliability.	Smart environment, multi-sensor data fusion, Dempster–Shafer theory.
Boucherit, M. S et al., (2017) [20]	Detect sensor faults in nonlinear systems through adaptive threshold estimation.	Threshold estimation for fault detection in nonlinear systems.	Reliable sensor fault identification.	Nonlinear systems, sensor fault detection, threshold estimation.
Chirayil, A et al., (2019) [22]	Review and analyze anomaly detection methods in Wireless Sensor Networks (WSNs).	Comprehensive survey of anomaly detection algorithms.	Overview of strengths and limitations.	Wireless Sensor Networks, anomaly detection techniques.
Yuan, K et al., (2016) [23]	Model sensor reliability to improve fault diagnosis accuracy using evidence theory.	Evidence-theory-based sensor reliability modeling.	Enhanced fault diagnosis precision.	Sensor reliability, fault diagnosis, evidence theory.
Ghosh, N et al., (2020) [24]	Detect faults in IoT-based applications using sensor data fusion techniques.	Dempster–Shafer theory of evidence for multi-sensor data fusion.	Effective fault detection in IoT.	IoT, sensor data fusion, Dempster–Shafer theory.

**Table 2:** Performance metrics experimented with different anomaly values.

Anomaly (%)	Failing Sensors	Precision	Recall	F1-Score	Accuracy
0	0	0.984920	0.9834	0.986800	0.9892
10	0	0.953182	0.8958	0.923600	0.9259
20	0	0.921415	0.8020	0.857571	0.8668
30	0	0.878713	0.7158	0.788934	0.8085
40	0	0.849026	0.6276	0.721711	0.7580

**Table 3:** Performance metrics with failed sensors.

Anomaly	Failing Sensors	Precision	Recall	F1-Score	Accuracy
0.0	0	0.984920	0.9834	0.986800	0.9892
0.0	1	0.951873	0.4024	0.573873	0.7012
0.0	2	0.953902	0.3962	0.567540	0.6981
0.0	3	0.928484	0.3640	0.533724	0.6820
0.0	4	0.883599	0.3552	0.524203	0.6776

## Data Availability

The data used are from online resources and simulated data used for verification.

## Abbreviations

The following abbreviations are used in this manuscript.

**Notations**	**Descriptions**
$\{S_{1}DA,S_{2}DA,\;S_{3}DA,S_{4}DA,$ $S_{5}DA\}$	Data acquision from RGB, IR, Gas, Smoke and Flame sensor
$\{S_{1}N_{i},S_{2}N_{i},S_{3}N_{i},$ ${\;S}_{4}N_{i}\;,S_{5}N_{i}\}$_._	Normalized the sensor raw values
$\left\{S_{1}M,\;S_{2}M,\;S_{3}M,\;S_{4}M,\;S_{5}M\right\}$	Mean of the Normalized data
{S1SD, S2SD, S2SD, S3SD, S4SD, S5SD}	Standard Deviation of the normalized data
{DynamicUpperThresholdi, DynamicLowerThresholdi}	For Anomaly detection.
Si_anomaly	record the anomaly detected for faulty sensor
$\{S_{1}AD,\;S_{2}AD,\;S_{3}AD,$ $S_{4}AD,S_{5}AD\}$	Anomaly detection of each sensor
DE(m_i_)	Deng Entropy
$\left\{ML_{1},\;ML_{2},\;ML_{3},\;ML_{4},\;ML_{5}\right\}$	Class probabilities prediction from each of 5 ML Models
CWF	Combined weight factor
PWC	Percentage of weight (%) for calculating combined weight factor
${m(fire)}_{K}$ ${m(no\;fire)}_{K}$	Probabilistic model adjustment based on sensor reliability
TotalConfidence	confidence score from all models (both normal and faulty sensors)
$m\left(fire\right)$ $\left(no\;fire\right)$ m(Θ+)	Belief mass for fire, Belief mass for No fire Belief mass for entire hypothesis
M_new_(fire)	Dynamically Belief
H(m_i_,m_j_)	Hellinger Distance
$\{S_{1}DP,\;S_{2}DP,\;S_{3}DP,$ $S_{4}DP,S_{5}DP\}$	Pre-processed sensor data
m_combined_(A)	Dempster’s Rule of Combination
S_1_RGB, S_2_IR, S_3_Gas, S_4_Smoke, S_5_Flame	Multi sensor data type
Belief(A), Plausibility(A)	Belief and Plausibility for each class

## References

[1] Nesa N., Banerjee I. (2017). IoT-Based sensor Data Fusion for occupancy sensing using Dempster–Shafer Evidence Theory for smart buildings. IEEE Internet Things J..

[2] Zervas E., Mpimpoudis A., Anagnostopoulos C., Sekkas O., Hadjiefthymiades S. (2009). Multisensor data fusion for fire detection. Inf. Fusion.

[3] Trapani N., Longo L. (2023). Fault Detection and Diagnosis Methods for Sensors Systems: A Scientific Literature Review. IFAC-Pap..

[4] Li F., Xie R., Wang Z., Guo L., Ye J., Ma P., Song W. (2019). Online distributed IoT security monitoring with multidimensional streaming big data. IEEE Internet Things J..

[5] Habbouche H., Benkedjouh T., Amirat Y., Benbouzid M. (2021). Gearbox failure diagnosis using a multisensor data-fusion machine learning-based approach. Entropy.

[6] Mellit A., Herrak O., Rus Casas C., Massi Pavan A. (2021). A machine learning and internet of things-based online fault diagnosis method for photovoltaic arrays. Sustainability.

[7] Rouzbahani H.M., Karimipour H., Lei L. An ensemble deep convolutional neural network model for electricity theft detection in smart grids. Proceedings of the 2020 IEEE International Conference on Systems, Man, and Cybernetics (SMC).

[8] De Paola A., Ferraro P., Gaglio S., Re G.L., Das S.K. (2016). An adaptive bayesian system for context-aware data fusion in smart environments. IEEE Trans. Mobile Comput..

[9] Hamda N.E.I., Hadjali A., Lagha M. (2023). Multisensor Data Fusion in IoT Environments in Dempster–Shafer Theory Setting: An Improved Evidence Distance-Based Approach. Sensors.

[10] Su Z., Wang F., Xiao H., Yu H., Dong S. (2020). A fault diagnosis model based on singular value manifold features, optimized SVMs and multi-sensor information fusion. Meas. Sci. Technol..

[11] Qiao S., Song B., Fan Y., Wang G. (2023). A Fuzzy Dempster–Shafer Evidence Theory Method with Belief Divergence for Unmanned Surface Vehicle Multi-Sensor Data Fusion. J. Mar. Sci. Eng..

[12] Li S., Liu G., Tang X., Lu J., Hu J. (2017). An Ensemble Deep Convolutional Neural Network Model with Improved D-S Evidence Fusion for Bearing Fault Diagnosis. Sensors.

[13] Wang Z., Xiao F. (2018). An improved multisensor data fusion method and its application in fault diagnosis. IEEE Access.

[14] Attarha S., Förster A. (2024). AssureSense: A framework for enabling sensor fault detection in Low-Power IoT edge devices. IEEE Sens. J..

[15] Wang Z., Wang R., Gao J., Gao Z., Liang Y. (2019). Fault recognition using an ensemble classifier based on Dempster–Shafer Theory. Pattern Recognit..

[16] Hui K.H., Lim M.H., Leong M.S., Al-Obaidi S.M. (2016). Dempster-Shafer evidence theory for multi-bearing faults diagnosis. Eng. Appl. Artif. Intell..

[17] Xiao F. (2020). Evidence combination based on prospect theory for multi-sensor data fusion. ISA Trans..

[18] Lin Y., Li Y., Yin X., Dou Z. (2018). Multisensor fault diagnosis modeling based on the evidence theory. IEEE Trans. Reliab..

[19] Ullah I., Youn J., Han Y. (2021). Multisensor data fusion based on Modified Belief Entropy in Dempster–Shafer Theory for smart Environment. IEEE Access.

[20] Boucherit M.S., Ladjouzi S., Kacimi N., Grouni S., Soufi Y. (2017). Sensor fault detection in nonlinear system using threshold estimation. Int. J. Digit. Signals Smart Syst..

[21] Mukhopadhyay S., Schurgers C., Panigrahi D., Dey S. (2008). Model-based techniques for data reliability in wireless sensor networks. IEEE Trans. Mobile Comput..

[22] Chirayil A., Maharjan R., Wu C. Survey on Anomaly Detection in Wireless Sensor Networks (WSNs). Proceedings of the 2019 20th IEEE/ACIS International Conference on Software Engineering, Artificial Intelligence, Networking and Parallel/Distributed Computing (SNPD).

[23] Yuan K., Xiao F., Fei L., Kang B., Deng Y. (2016). Modeling sensor reliability in fault diagnosis based on evidence theory. Sensors.

[24] Ghosh N., Paul R., Maity S., Maity K., Saha S. (2020). Fault Matters: Sensor data fusion for detection of faults using Dempster–Shafer theory of evidence in IoT-based applications. Expert Syst. Appl..

[25] Luo W.B., Caselton B. (1997). Using Dempster–Shafer theory to represent climate change uncertainties. J. Environ. Manag..

[26] Dinh H.T., Lee C., Niyato D., Wang P. (2013). A survey of mobile cloud computing: Architecture, applications, and approaches. Wirel. Commun. Mob. Comput..

[27] Kumar A.S., Raja S., Pritha N., Raviraj H., Lincy R.B., Rubia J.J. (2022). An adaptive transformer model for anomaly detection in wireless sensor networks in real-time. Meas. Sens..

[28] Zhao K., Li L., Chen Z., Sun R., Yuan G., Li J. (2022). A survey: Optimization and applications of evidence fusion algorithm based on Dempster–Shafer theory. Appl. Soft Comput..

[29] Xia J., Feng Y., Liu L., Liu D., Fei L. (2018). An Evidential Reliability Indicator-Based Fusion Rule for Dempster-Shafer Theory and its Applications in Classification. IEEE Access.

[30] Song Y., Deng Y. (2019). Divergence measure of belief function and its application in data fusion. IEEE Access.

[31] Gaddam A., Wilkin T., Angelova M., Gaddam J. (2020). Detecting sensor faults, anomalies and outliers in the internet of Things: A survey on the challenges and solutions. Electronics.

[32] Zhou J., Hu L., Wang F., Lu H., Zhao K. (2013). An efficient multidimensional fusion algorithm for IoT data based on partitioning. Tsinghua Sci. Technol..

[33] Gao J., Jia D., Hu N. (2024). Improved random forest model for smoke detection and early warning based on sparrow search algorithm. Appl. Comput. Eng..

[34] Yang L., Shami A. (2022). IoT data analytics in dynamic environments: From an automated machine learning perspective. Eng. Appl. Artif. Intell..

[35] Kiranyaz S., Gastli A., Ben-Brahim L., Al-Emadi N., Gabbouj M. (2018). Real-Time fault detection and identification for MMC using 1-D convolutional neural networks. IEEE Trans. Ind. Electron..

[36] Jeong Y., Hwang J., Lee S., Ndomba G.E., Kim Y., Kim J. (2024). Sensor-Based indoor fire forecasting using transformer Encoder. Sensors.

[37] Zhang Z., Jiang W., Geng J., Deng X., Li X. (2020). Fault diagnosis based on Non-Negative Sparse Constrained Deep Neural Networks and Dempster–Shafer theory. IEEE Access.

[38] Wang H., Shi Y., Chen L., Zhang X. (2024). A Tunnel Fire Detection Method Based on an Improved Dempster-Shafer Evidence Theory. Sensors.

[39] Munz M., Dietmayer K. Using Dempster-Shafer-based modeling of object existence evidence in sensor fusion systems for advanced driver assistance systems. Proceedings of the 2011 IEEE Intelligent Vehicles Symposium (IV).

[40] Li N.M., Tao N.Z. (2016). Health status identification of rolling bearing based on SVM and improved evidence theory. Equip. Health Status Identif. SVM. D-S Evid. Theory Datafusion Multi-Sens..

[41] Khan M.N., Anwar S. (2019). Paradox Elimination in Dempster–Shafer Combination Rule with Novel Entropy Function: Application in Decision-Level Multi-Sensor Fusion. Sensors.

[42] Okafor N. (2023). Advances and Challenges in IoT Sensors Data Handling and Processing in Environmental Monitoring Systems. TechRxiv.

[43] Gui W., Lu Q., Su M., Pan F. (2020). Wireless Sensor Network Fault Sensor Recognition Algorithm Based on MM* Diagnostic Model. IEEE Access.

[44] Seiti H., Hafezalkotob A. (2018). Developing pessimistic–optimistic risk-based methods for multi-sensor fusion: An interval-valued evidence theory approach. Appl. Soft Comput..

[45] Yager R.R. (1987). On the dempster-shafer framework and new combination rules. Inf. Sci..

[46] Senouci M.R., Mellouk A., Aitsaadi N., Oukhellou L. (2016). Fusion-based surveillance WSN deployment using Dempster–Shafer theory. J. Netw. Comput. Appl..

[47] Azar J., Makhoul A., Barhamgi M., Couturier R. (2019). An energy efficient IoT data compression approach for edge machine learning. Future Gener. Comput. Syst..

[48] Sharma M., Maity T. (2022). Multisensor Data-Fusion-Based Gas Hazard Prediction using DSET and 1DCNN for underground Longwall coal Mine. IEEE Internet Things J..

[49] Sung S., Hong S., Choi H., Park D., Kim S. (2024). Enhancing Fault Diagnosis in IoT Sensor Data through Advanced Preprocessing Techniques. Electronics.

[50] Çavdar T., Ebrahimpour N., Kakız M.T., Günay F.B. (2022). Decision-making for the anomalies in IIoTs based on 1D convolutional neural networks and Dempster–Shafer theory (DS-1DCNN). J. Supercomput..

[51] Sharma M., Maity T. (2023). Smart and Fault Tolerant Multi-Sensor Fusion Model for UCM Methane Hazard Monitoring Based on Belief Divergence Backed DS Filter and Hybrid CNN-LSTM Classifier. IEEE Internet Things J..

[52] Kumar M., Singh S.K., Kim S. (2025). Hybrid deep learning-based cyberthreat detection and IoMT data authentication model in smart healthcare. Future Gener. Comput. Syst..

[53] Singh S.K., Kumar M., Khanna A., Virdee B. (2024). Blockchain and FL-based secure architecture for enhanced external Intrusion detection in smart farming. IEEE Internet Things J..

[54] Kumar M., Kim C., Son Y., Singh S.K., Kim S. (2024). Empowering cyberattack identification in IoHT networks with neighborhood component-based improvised long short-term memory. IEEE Internet Things J..

